# Satellite-based monitoring of forage quality in grasslands of the United Kingdom using sentinel-2 data and random forest regression

**DOI:** 10.3389/fvets.2025.1678123

**Published:** 2025-12-19

**Authors:** J. G. N. Irisarri, M. A. Texeira, P. Harris, K. G. Pembleton

**Affiliations:** 1 Department of Ecosystem Science and Management, University of Wyoming, Laramie, WY, United States; 2 Laboratorio de Análisis Regional y Teledetección (LART), Instituto de Investigaciones Fisiológicas y Ecológicas Vinculadas a la Agricultura (IFEVA), Departamento de Métodos Cuantitativos y Sistemas de Información, Facultad de Agronomía, Universidad de Buenos Aires, Buenos Aires, Argentina; 3 Rothamsted Research North Wyke, Okehampton, United Kingdom; 4 Centre for Sustainable Agricultural Systems, University of Southern Queensland, Toowoomba, QLD, Australia

**Keywords:** sentinel-2, North Wyke farm platform, grassland, crude protein, NDF, ADF, water soluble content

## Abstract

**Introduction:**

In the temperate grasslands of the UK, forage quality is a key factor influencing both animal performance and environmental impact. Because forage quality strongly affects rumen fermentation, improving it can reduce enteric methane emissions and mitigate animal nutritional stress. However, large-scale monitoring of forage quality remains limited due to the reliance on destructive, labor-intensive, and costly sampling methods. Remote sensing offers a promising alternative for scalable monitoring.

**Methods:**

We explored an indicative approach combining optical remote sensing (Sentinel-2) with random forest regression (RFR) models to predict four critical forage quality attributes: crude protein (CP), water-soluble carbohydrates (WSC), neutral detergent fiber (NDF), and acid detergent fiber (ADF). Calibration and validation were performed using >9,500 georeferenced observations collected between 2020 and 2022 at the North Wyke Farm Platform in southwest UK. Forage quality was measured using near-infrared (NIR) sensors mounted on agricultural machinery across paddocks containing permanent and improved pastures. Sentinel-2 spectral predictors included visible, NIR, and red-edge bands, and model performance was evaluated using R² and RMSE metrics.

**Results:**

Model performance was strong across all four forage quality attributes, with R² values ranging from 0.77 to 0.86 and consistently low RMSE values, indicating high predictive accuracy. Red-edge and NIR wavelengths were the most influential predictors. Improved pastures generally exhibited higher forage quality—characterized by lower ADF and higher WSC concentrations—than permanent pastures. Model-predicted seasonal changes were modest, whereas spatial contrasts between paddocks were much more pronounced.

**Discussion:**

The calibrated models are suitable for forage systems with species composition and quality ranges similar to those represented in our dataset but should not be directly applied to other forage types without recalibration. Overall, this work demonstrates the potential of Sentinel-2 remote sensing combined with machine-learning approaches for tolerably accurate, large-scale forage quality monitoring. Such advancements could help improve grazing management, support nutritional planning, and contribute to efforts aimed at reducing methane emissions from livestock systems.

## 1 Introduction

In temperate regions worldwide, grazing-based livestock production supplies a substantial share of high-quality animal protein and dairy products (1). Forage quality is a key determinant of animal productivity and the environmental footprint (2, 3). In the face of climate change, temperate grasslands are emerging as crucial for food security. Their unique ability to utilize fibrous materials enables sustainable production where other systems may struggle. Detailed monitoring of forage quality dynamics is thus essential to enhance animal performance while minimizing environmental impacts (4–7). This study focuses on temperate grasslands in the United Kingdom, providing a context where pasture-based livestock production is predominant and forage quality monitoring is critical for sustainable management. This requires addressing a fundamental challenge in grasslands—capturing changes in key forage quality attributes across both time and space with high frequency.

At least four forage quality attributes can be considered critical for animal performance and environmental outcomes: crude protein (CP), water-soluble carbohydrates (WSC), and the fiber fractions neutral detergent fiber (NDF) and acid detergent fiber (ADF). Optimal CP levels enhance feed conversion efficiency via rumen microbial synthesis, while excess CP leads to nitrous oxide (N₂O) emissions and nitrate leaching; mismatches with WSC can also increase methane (CH₄) production through inefficient fermentation (8–12). Several authors (8–12) showed that high WSC levels enhance fiber digestibility, leading to greater weight gain and milk production. In contrast, high WSC levels enhance fiber digestibility, leading to greater weight gain and milk production. These authors (8–12) also found that WSC-rich diets reduce the acetate:propionate ratio in rumen fermentation, favoring propionate, a less methanogenic pathway and potentially lowering enteric CH₄ emissions per unit of animal product. NDF represents the structural fraction of the plant that limits voluntary dry matter (DM) intake. Its digestibility depends on lignin content and microbial activity in the rumen (13, 14). Moderate NDF levels are necessary to maintain rumen motility, but high levels reduce intake and passage rate, limiting animal performance. ADF includes the least digestible forage components (cellulose and lignin) and is a direct predictor of DM digestibility. High ADF levels reduce overall digestibility, lower energy availability, and increase CH_4_ emissions per unit of metabolizable energy (13). Indeed, digestibility metrics such as organic matter digestibility (OMD) or dry matter digestibility (DMD) are often derived from ADF and NDF values, highlighting the integrative role of these attributes in estimating overall forage quality.

Traditional prediction of these attributes relies on destructive, costly lab analysis with limited scale (15). Machinery-mounted NIR sensors provide high-resolution alternatives (≈1.5 m) during harvests. One such system is the John Deere HarvestLab™ 3,000, which performs real-time NIR-based measurements of forage constituents during harvest. It operates using calibration curves developed in laboratory settings, matched to the reflectance spectra of the crop material. The system is widely used in commercial agriculture and has been certified for accuracy in dry matter estimation by independent organizations such as the Deutsche Landwirtschafts-Gesellschaft, DLG. Sentinel-2 satellite observations, from the visible, red-edge and NIR bands, show strong potential as predictors of forage biochemistry (6, 16–21). Spectral bands in the visible, red, red-edge, and NIR regions (bands 2, 3, 4, 5, 6, 7, 8, and 8A) capture variations in canopy structure and composition, which can influence livestock post-ingestive behavioral responses. However, calibrating and validating satellite observations requires reliable and representative reference data.

This study aims to calibrate and validate random forest regression (RFR) models for predicting four key forage quality attributes (CP, WSC, ADF, and NDF) using NIR-harvester data. It also identifies key Sentinel-2 spectral portions for predictions, enabling better grazing decisions that enhance animal welfare by reducing nutritional stress. Models were developed for the agroecological conditions of southwest UK, trained with 70% of the available data and validated with the remaining 30%, using an optimized RFR configuration. This integrated approach is designed to support accurate and cost-effective forage monitoring, with potential applications in pasture management, forage harvest planning, and precision livestock nutrition.

## 2 Materials and methods

### 2.1 Ethics statement

No animal studies are presented in this manuscript. No ethical clearance was required as the study did not involve direct animal experimentation.

### 2.2 Site

The study was conducted at the North Wyke Farm Platform (NWFP), located in southwest England, UK (50°46′12″N, 3°54′05″W), at an elevation ranging from 120 to 190 meters above sea level (22). The region has a temperate humid climate, with an average annual precipitation of 1,031 mm (range: 705–1,361 mm) and average daily minimum and maximum temperatures of 6.8 °C and 13.5 °C, respectively, for the period 1982–2019 (23). The predominant soils belong to the Hallsworth (Dystric Gleysol) and Halstow (Gleyic Cambisol) series, characterized by a moderately stony clay loam surface layer (~36% clay) over a denser mottled clay subsoil (~60% clay), derived from Carboniferous rocks (23).

The NWFP comprises 63 ha with contrasting pasture treatments: permanent (Green farmlet) and improved (Blue farmlet) pastures, representing typical temperate livestock systems (24). The study focused on data from 2020–2022 (24). In our analysis, the forage quality data used for RFR calibration and validation came exclusively from the two pasture types within the Green and Blue farmlets—permanent pastures dominated by *Lolium perenne L.* (Green) and improved pastures composed of *Lolium perenne L.* and *Trifolium repens L.* (Blue). These pasture systems represent typical livestock production systems in temperate environments (24).

### 2.3 Data

Ground reference data were obtained from a NIR sensor mounted on a John Deere forage harvester, while the remote spectral data were derived from Sentinel-2 satellite imagery. The NIRS sensor used in this study was the John Deere HarvestLab™ 3,000, which employs proprietary calibration models developed from thousands of laboratory-analyzed forage samples. These calibrations are embedded in the device and benchmarked against wet-chemical reference methods, as validated by independent testing (DLG, calibration model LKS 04/18).^1^ The sensor reports forage constituents including dry matter, crude protein (CP), neutral detergent fiber (NDF), acid detergent fiber (ADF), and water-soluble carbohydrates (WSC), primarily on a dry matter basis. Moisture content is also measured directly, allowing conversion to fresh matter values if needed. While the calibration specifications (e.g., precision, sensitivity) are proprietary, the sensor has been validated for field use and shows performance comparable to laboratory standards. These calibrations were used to generate reference values for model training and evaluation. The sensor operates in the NIRS range (1,100–2,500 nm) and is integrated into the harvester’s monitoring system, allowing data to be recorded at an approximate spatial resolution of 1.5 meters in the direction of travel. This enables detailed characterization of within-paddock variability.

The forage quality data for the NWFP’s Green and Blue farmlets were collected during the 2020, 2021, and 2022 harvest campaigns, at two points in the year: a first harvest in late May or early June, and a second in mid to late August. All sampled paddocks were managed for silage production during the study period. No grazing occurred prior to harvest, ensuring that biomass accumulation reflected ungrazed growth. This management context is relevant for interpreting forage composition, as it tends to result in higher biomass and more uniform canopy structure compared to grazed systems. Data were automatically recorded by the harvester’s integrated system, which includes high-precision GPS, and later downloaded from the John Deere online platform. Each harvester observation represented an approximate area of 4–5 m^2^, based on a 1.5 m resolution in the direction of travel and the width of the cutting head. Geolocation accuracy was ensured through triangulation with a local ground station at the North Wyke head office, providing sub-meter precision for all georeferenced points. For data analysis, the data were exported in shapefile format. A cleaning process was applied, including the removal of observations with missing values for any attribute and the exclusion of outliers identified through variable relationship inspection. NIRS measurements were performed post-harvest using a sensor mounted on a forage harvester. Therefore, the spectral data used for calibration represent mowed biomass rather than canopy-level estimates.

Sentinel-2 data were extracted via Google Earth Engine (25), selecting the closest low-cloud image (<20% cover, typically within 8 ± 5 days prior, range = 3–13 days) for each NIR point. Bands B2–B8A and B11 were used. The extraction was performed via point sampling on each selected image, and the spectral values were linked to each forage quality sampling point from the harvester. This procedure enabled the association of satellite spectral data with high-resolution ground reference data, facilitating the calibration of forage quality prediction models. Each Sentinel-2 pixel typically aggregated between 12 to 14 NIR observations.

### 2.4 Data analysis

**Figure 1:**
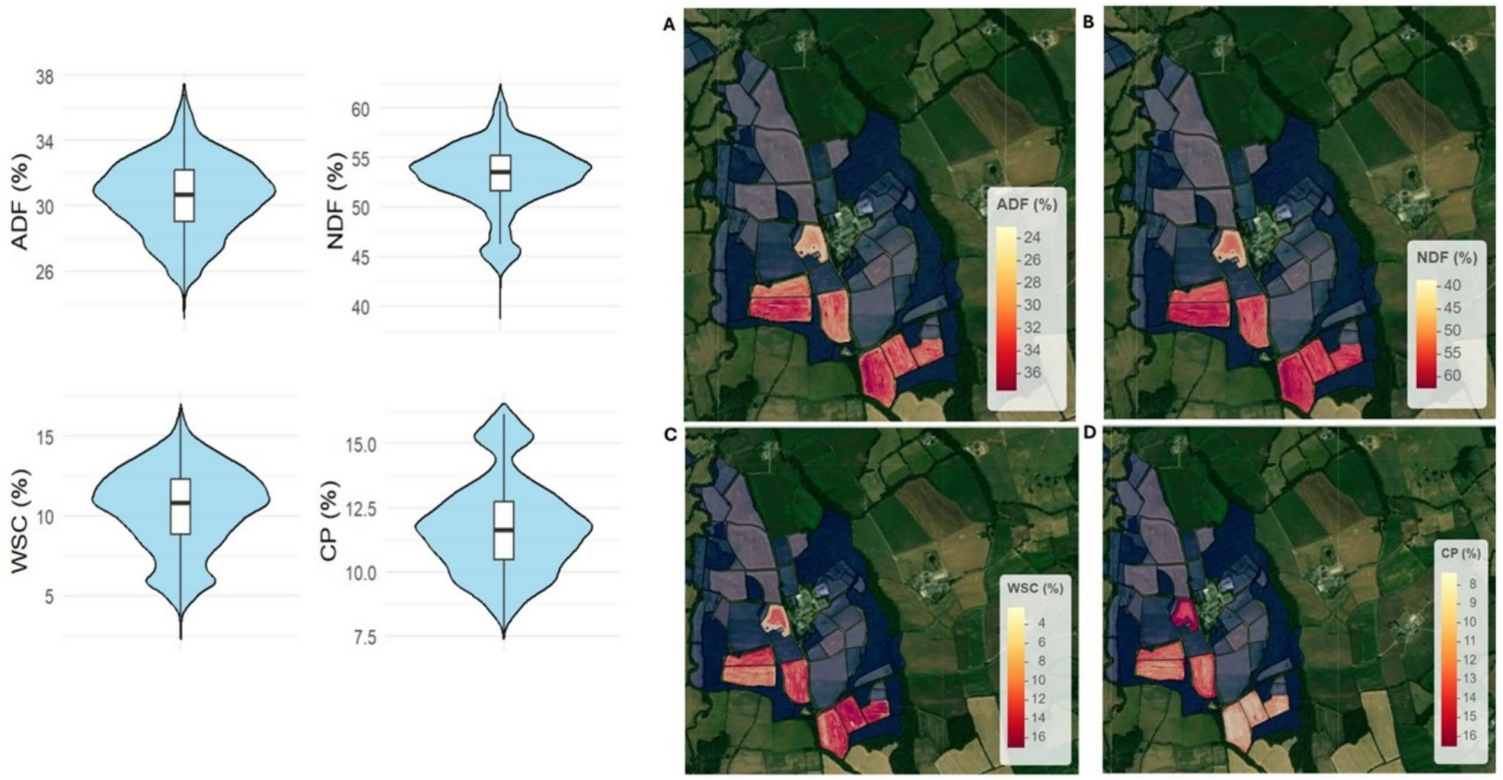
Non-spatial and spatial distributions of the four key forage quality attributes predicted using near-infrared (NIR) sensors mounted on farm machinery (harvesters) by the John Deere system, at six paddocks of the North Wyke Farm Platform (NWFP). The violin plots (left) show the distribution of values for: **(A)** acid detergent fiber (ADF), **(B)** neutral detergent fiber (NDF), **(C)** Sugar (water-soluble carbohydrates), and **(D)** crude protein (CP). The maps (right) display the spatial variation of each attribute both within and across paddocks. Warm colors indicate higher values. Maps represent the aggregation of all NIRS measurements collected between 2020 and 2023, across multiple sampling events. All values are expressed on a dry matter basis.

To predict forage quality attributes from satellite data, RFR models were fitted using functions available in the `randomForest` package in R. Independent RFRs were developed for each of the four attributes: CP, WSC, NDF and ADF. The dataset included a total of 9,531 observations, which were randomly split into 70% for training and 30% for validation. The predictor variables included the optical bands from Sentinel-2 (B2, B3, B4, B5, B6, B7, B8, and B8A). Each RFR was trained using 2,000 trees and five randomly selected predictors (mtry = 5) at each split, values determined through manual tuning that optimized predictive accuracy and model stability. The relative importance of each predictor variable was assessed using the mean decrease in the accuracy metric and visualized through bar plots. Model validation was performed on the test dataset by comparing predicted values with observed values using scatter plots and calculating the Pearson correlation coefficient (r). Hyperparameter tuning was performed manually, without formal grid or random search. Multiple configurations were evaluated heuristically, and the final model parameters were selected based on performance on independent test datasets. In addition to Pearson correlation, we calculated R^2^, RMSE, and MAE to provide a more comprehensive evaluation of model accuracy and generalization, as shown in Figure 1.

## 3 Results

**Figure 2:**
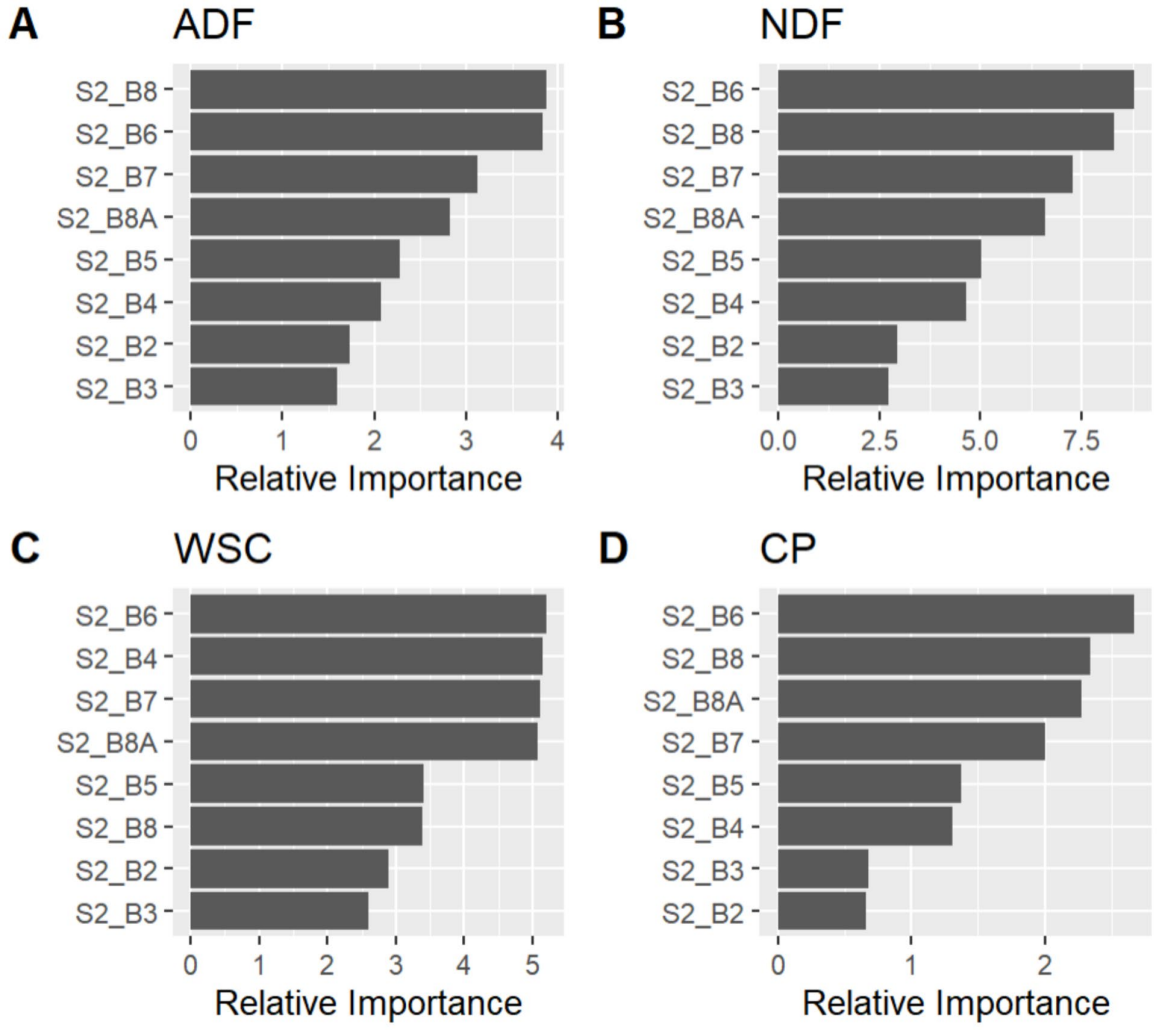
Relative (variable) importance of different Sentinel 2 spectral bands for the random forest regression (RFR) prediction of four forage quality components: **(A)** Acid Detergent Fiber (ADF), **(B)** Neutral Detergent Fiber (NDF), **(C)** Water-Soluble Carbohydrates (WSC), and **(D)** Crude Protein (CP).

The four forage quality attributes predicted by the John Deere system showed marked variability across the six NWFP paddocks (Figure 2). WSC ranged fourfold (4–16%), CP twofold (8–16%), NDF 1.5-fold (40–60%), and ADF 1.4-fold (26–36%). These datasets provided the target variables for the RFR models. These datasets, reflecting potential shifts in stem-to-leaf ratios and selectivity in grazing, provided the target variables for RFR models.

**Figure 3:**
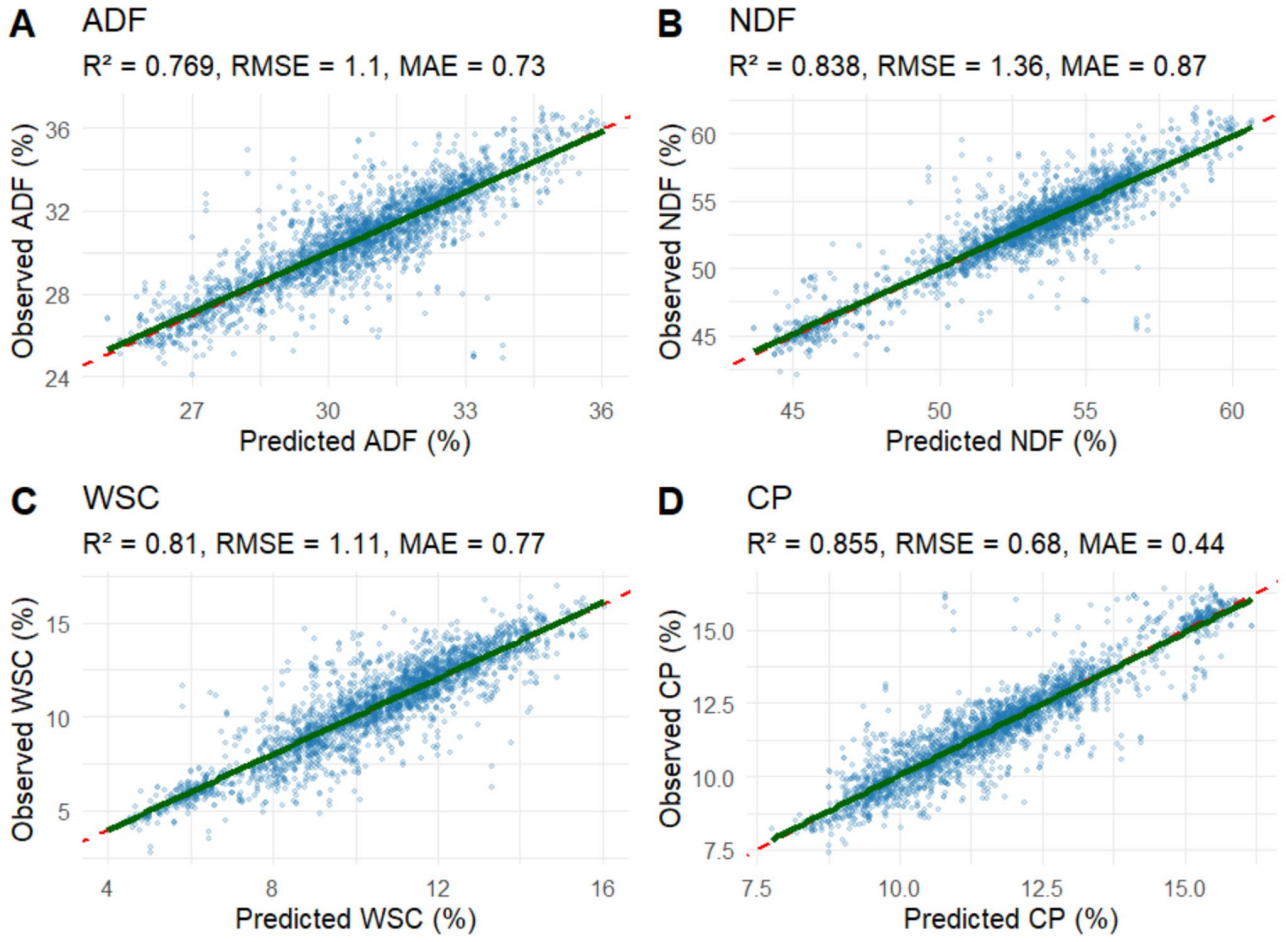
Relationship between observed (John Deere predicted) and random forest regression (RFR) (satellite-informed) predicted values for four forage quality attributes: **(A)** Acid Detergent Fiber (ADF), **(B)** Neutral Detergent Fiber (NDF), **(C)** Water-Soluble Carbohydrates (WSC), and **(D)** Crude Protein (CP). Each panel shows a scatter plot with the Pearson correlation coefficient (r) between observed values (y-axis) and values predicted by the RFRs (x-axis), using independent validation data.

For the fitted Random Forest Regression (RFR) models, Sentinel-2 NIR (S2_B8), red-edge (S2_B6, B7, B8A), and visible (S2_B4) bands were most relevant. SWIR band B11 was not influential, possibly due to limited moisture variability in this system (despite literature support in drier regions), as shown by the variable importance plots (Figure 3). In the case of ADF, the red-edge bands (S2_B6, B7) and narrow NIR (S2_B8A) contributed most to the model’s performance. For NDF, a combination of red-edge and NIR bands were the most influential. For WSC (sugars), the red-edge band S2_B6 and the red band (S2_B4, in the visible spectrum) dominated in importance, indicating a strong relationship between these wavelengths and soluble carbohydrate content. Finally, for CP (crude protein), variable importance was more evenly distributed among visible (S2_B4), red-edge (S2_B6), and NIR (S2_B8) bands, suggesting greater spectral complexity in its prediction using RFR.

**Figure 4:**
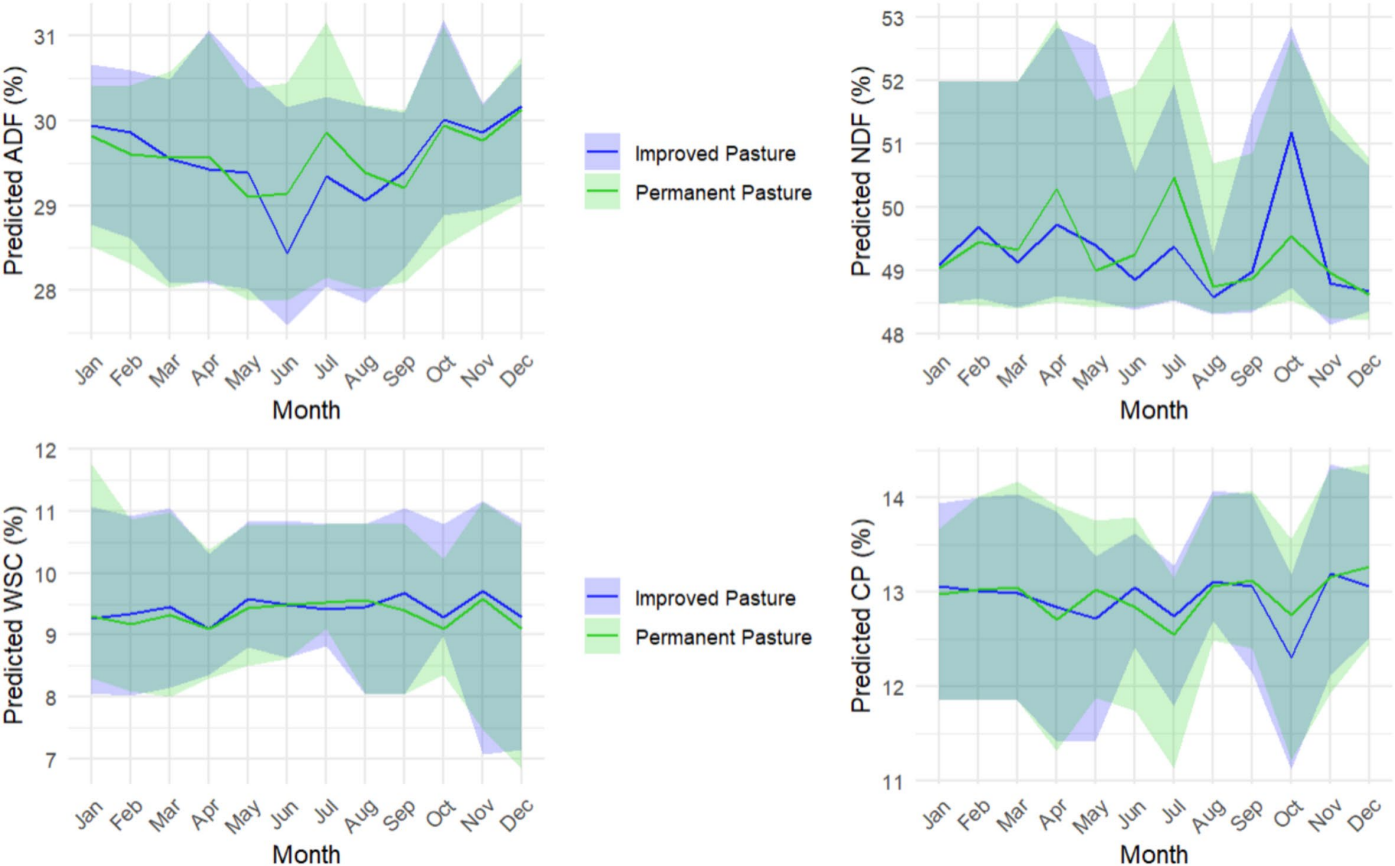
Seasonal dynamics of forage quality attributes predicted by Random Forest Regression (RFR) models in two functional pasture communities: permanent and improved pastures. Median monthly values are shown for **(A)** Acid Detergent Fiber (ADF), **(B)** Neutral Detergent Fiber (NDF), **(C)** Water-Soluble Carbohydrates (WSC), and **(D)** Crude Protein (CP). Lines represent the monthly medians, and shaded areas denote the interquartile range, summarizing the spatial and temporal variability in model predictions across the study area. Values integrate the 2020–2022 period, and the RFR models were trained using bi-seasonal field data collected during those years, capturing spatial, seasonal, and interannual variability in forage quality relevant to ruminant nutrition.

The study RFRs, informed by satellite data, showed high predictive power for all four forage quality attributes as indicated by the performance metrics (Figure 4). Among traits, CP exhibited the best overall performance (R^2^ = 0.86, RMSE = 0.68, MAE = 0.44), followed by WSC (R^2^ = 0.81, RMSE = 1.11, MAE = 0.77) and NDF (R^2^ = 0.84, RMSE = 1.36, MAE = 0.87). ADF predictions were also robust (R^2^ = 0.77, RMSE = 1.10, MAE = 0.73), though slightly more dispersed at extreme values. Specifically, the RMSE values indicate typical prediction errors of approximately 0.7 percentage points for CP, 1.1 for WSC, 1.1 for ADF, and 1.4 for NDF. These error levels represent <10% of the observed range for each attribute, confirming the models’ strong predictive accuracy for practical forage quality monitoring. All Random Forest model files (.rds) and accompanying documentation are available through the Zenodo repository.^2^

Seasonal dynamics of forage quality attributes predicted by the RFR models revealed distinct patterns between the two functional communities, namely the permanent and improved pastures of the NWFP (Figure 1). ADF concentration followed a U-shaped pattern, with minimum values around mid-year and peaks at the beginning and end of the year. In improved pastures, lower ADF levels were maintained from May to August, indicating better forage quality for this structural component. NDF also showed noticeable seasonal fluctuations, with peaks in April, July, and October, and a gradual decline toward the end of the year. Permanent pastures exhibited slightly higher NDF values for most of the year, except during the October peak. In contrast, WSC and CP showed limited seasonal variation, remaining relatively stable throughout the year. Across all attributes, the shaded interquartile ranges revealed substantial temporal and spatial variability, particularly for ADF and NDF during spring and early summer, reflecting heterogeneous canopy structures and phenological stages among fields. Conversely, WSC and CP displayed narrower variability bands during mid-summer, suggesting more uniform forage quality at the peak of vegetative growth.

**Figure 5:**
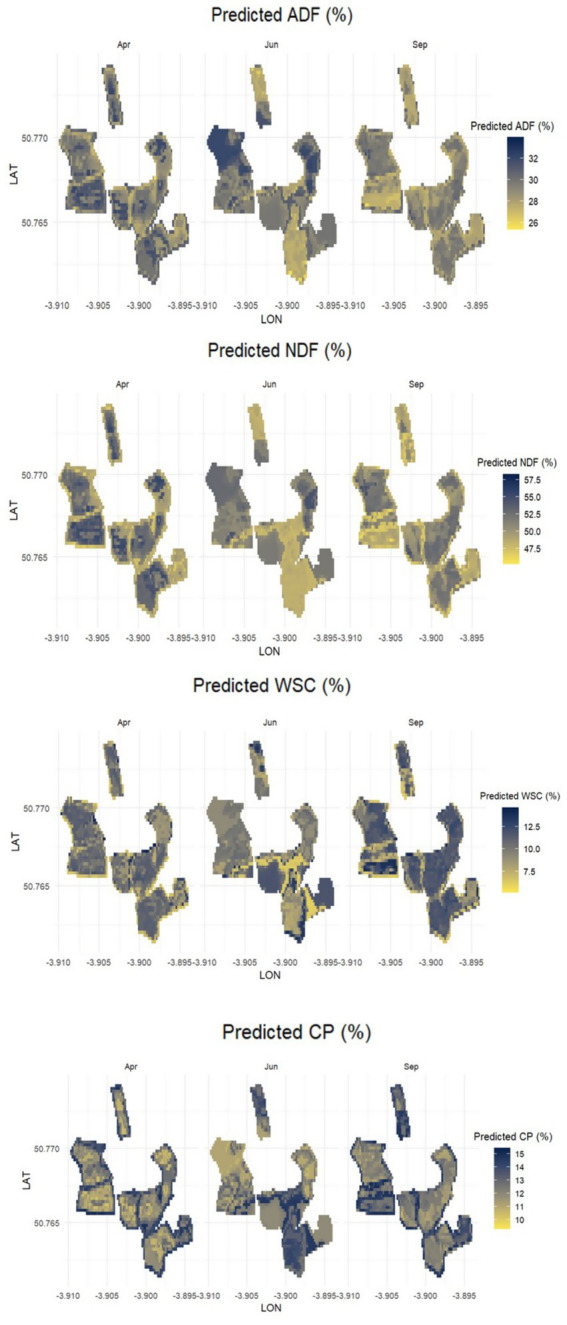
Spatial patterns of forage quality attributes predicted by Random Forest Regression (RFR) models derived from Sentinel-2 spectral data across the study area. Mean predictions for Acid Detergent Fiber (ADF), Neutral Detergent Fiber (NDF), Water-Soluble Carbohydrates (WSC), and Crude Protein (CP) are shown for April, June, and September, integrating the 2020–2022 period. Each map represents the spatial distribution of model-predicted values averaged per month and attribute, highlighting spatial heterogeneity and seasonal shifts in forage quality. The RFR models were trained using bi-seasonal field data collected during 2020–2022, allowing the predictions to capture both spatial and temporal variation relevant to ruminant nutrition.

Spatial patterns of predicted forage quality attributes across the study area showed marked heterogeneity among fields and greater than seasonal shifts (Figure 5). Spatial variability was primarily expressed across fields rather than within them, suggesting that differences in management and vegetation composition were main drivers of spatial heterogeneity in predicted quality attributes. This field-level variability remained consistent throughout the months, underscoring the ability of Sentinel-2 spectral data combined with RFR modeling to detect management-driven contrasts in forage quality across the landscape. Seasonal variation suggested that in April, higher ADF and NDF values were concentrated in the central and northern sectors of the study area. By June, both structural components declined, reflecting the mid-season increase in forage quality. In contrast, WSC and CP exhibited spatially variable but generally higher values in June, coinciding with the peak of the growing season. In September, ADF and NDF increased again, while WSC and CP declined, indicating a late-season reduction in forage quality.

## 4 Discussion

Sentinel-2 data combined with Random Forest regression (RFR) accurately predicted the four key forage quality attributes (CP, WSC, ADF, and NDF), achieving validation statistics comparable to those reported in previous studies (17–21). The novelty of this work lies in the integration of satellite data with near-infrared reference information collected directly from a machinery-mounted NIRS system, enabling large-scale calibration under operational farm conditions. This highlights Sentinel-2’s robustness for estimating forage quality in grasslands of the United Kingdom, consistent with results observed in other temperate regions (20, 21). The approach is therefore applicable to forage systems with species composition and quality attribute ranges like those represented in our dataset. Extrapolation to environments with markedly different seasonal dynamics or forage types—such as those where crude protein regularly falls below 6%—would exceed the calibration range and is not recommended.

The variable importance analysis revealed that red-edge, near-infrared (NIR), and shortwave infrared (SWIR) bands (B5, B6, B7, B11, and B12) were the most influential for estimating the four forage quality attributes. This pattern is consistent with previous studies emphasizing the relevance of these spectral regions for assessing structural and chemical properties of vegetation, including lignin, cellulose, and soluble carbohydrates (20, 21). In particular, the red-edge band B7 showed the highest contribution, supporting earlier findings that identify it as a key predictor of dry matter and nutrient content (21). The combined role of red-edge and SWIR wavelengths enhances the prediction of both biomass and forage quality by reducing reflectance saturation in dense canopies (19, 20).

The seasonal dynamics of forage quality attributes showed very small amplitudes (<1 percentage point) compared to the variability observed during calibration (e.g., ADF ranging from 13 to 57% or CP from 6 to 36%). This low variability suggests that forage quality in this system is highly stable throughout the year, despite phenological changes. However, the spatial patterns revealed that fine-scale variability in forage quality across fields exceeded the average intra-annual variation observed within individual pastures. Such strong spatial contrasts likely reflect underlying heterogeneity in management and potentially factors associated with topographic positioning, clearly associated with SOC (26). This pattern is like that observed under similar pastures composition in Southern Australia (27). Moreover, the average values of ADF (~29%), NDF (~50%), and CP (~13%) fall within the upper range compared to global literature, where high-quality forages typically present ADF < 35%, NDF < 50%, and CP between 8–18% (28). Rather than implying broad management recommendations, we emphasize that spatially explicit information systems like this can support adaptive grazing strategies, for example by identifying areas of higher forage quality instead of relying on fixed rotations. It should be noted that our dataset does not include very low biomass levels typical of heavily grazed swards, such conditions often correspond to younger tissues with higher nutritive value, meaning that excluding them could lead to a slight underestimation of forage quality in contexts where quality is already high globally (28). Although improved pastures showed slightly higher forage quality values, the differences were modest. This reflects the fact that the primary goal of the improved pasture system is not only to enhance nutritional value, but to reduce nitrogen inputs and improve environmental sustainability (29).

Beyond decision-support for grazing management, the availability of spatially explicit nutritional information also offers significant value for improving greenhouse gas (GHG) estimation frameworks in pasture-based systems. First, remote estimates of crude protein can support a better understanding of individual dry matter intake as a percentage of live weight, a key determinant of enteric methane production under Tier 2 methodologies (30). Second, forage digestibility directly influences methane emissions per unit of dry matter intake, thus defining emission intensity (g CH₄ kg^−1^ DMI). While our models do not predict digestibility directly, digestibility (%) can be derived from the predicted ADF and CP using established nutritional frameworks, such as the Digestibility (Van Soest) model (31) or the Digestibility (Dairy model) tailored to temperate production systems (32, 33). By combining satellite-predicted forage constituents with physiologically grounded digestibility equations, this approach has the potential to provide more realistic and spatially refined estimates of enteric methane emissions, thereby supporting climate-smart grazing strategies.

Although the satellite-based models presented here performed well within the conditions of this study, their transferability to other pasture systems must be approached with caution. Machine learning approaches such as Random Forest rely on the coverage of the calibration domain: predictions can become unreliable when applied to forage conditions, botanical compositions, or management regimes that fall outside the biophysical space represented in the training data (34, 35). In our case, the observed ADF, NDF, and CP ranges align closely with those reported for temperate British pasture systems through national initiative known as GrassCheck GB (36), which supports confidence in our predictions. Nonetheless, broader adoption would require local calibration datasets to ensure validity under different livestock species, fertilization regimes, and climatic contexts. This highlights an important limitation of the current work but also a clear pathway toward operational scalability.

## 5 Conclusion

This study demonstrates the feasibility of integrating NIRS-derived forage quality data with Sentinel-2 spectral information to model and map key nutritional attributes of pastures at landscape scale. By combining multi-year and bi-seasonal field measurements with machine learning techniques, we provide a framework for predicting crude protein, fiber fractions, and sugar content with high spatial and temporal resolution. These findings offer a scalable and cost-effective approach for satellite-based forage monitoring, with direct relevance to temperate humid agricultural pastures. The models developed here can support decision-making in pasture management, forage harvest planning, and precision livestock nutrition. More broadly, this work contributes to the advancement of remote sensing applications in Rangeland and Grassland ecology, enabling researchers and producers to better assess forage dynamics and nutritional value across space and time. The applicability of this approach is limited to forage systems with species composition and quality ranges like those represented in our dataset; extrapolation to markedly different environments would require additional calibration data.

## Data Availability

The original contributions presented in the study are included in the article/supplementary material, further inquiries can be directed to the corresponding author.

## Footnotes

1
https://www.dlg.org/en/tests/agricultural-technology-and-farm-inputs/test-reports/test-john-deere-harvestlab-3000-kalibrationsmodell-lks04-18

2
https://doi.org/10.5281/zenodo.17543884
